# A Safe Approach to Percutaneous Tracheostomy for COVID-19 Patients in Intensive Care

**DOI:** 10.7759/cureus.14663

**Published:** 2021-04-24

**Authors:** Menka Chachlani, Mohammad Misurati, Karan Jolly, Ebrahim Ahmad, Michael Bright

**Affiliations:** 1 Critical Care Directorate, New Cross Hospital, Wolverhampton, GBR; 2 Critical Care Directorate, Royal Wolverhampton NHS Trust, Wolverhampton, GBR; 3 Orolaryngology, Royal Wolverhampton NHS Trust, Wolverhampton, GBR

**Keywords:** pulmonary critical care, difficult airway management, healthcare worker safety, ent procedures, percutaneous tracheostomy, covid-19

## Abstract

The novel coronavirus disease 2019 (COVID-19) has placed a burden on critical care facilities worldwide. Patients who remain critically unwell with COVID-19 require prolonged periods of ventilation, and the burden of both the resources during a pandemic and the slow respiratory wean must be managed. Percutaneous tracheostomies are commonplace in long-term intensive care patients, yet little is known about their role in COVID-19, particularly how operator safety is maintained during the procedure.

Here, we describe an approach designed to minimize cross-infection of the operators undertaking percutaneous tracheostomies within this subset of patients. Focus should be on non-technical skills, prolonged periods of pre-oxygenation, and minimal ventilation during the procedure to minimize the risk of aerosolization generated from an open breathing system. Our modified technique demonstrates successful early experiences with no operators testing positive for COVID-19 or developing symptoms following any performed procedure.

## Introduction

The recent pandemic caused by the novel coronavirus disease 2019 (COVID-19) has placed a large burden on healthcare systems worldwide. In particular, intensive care units (ICU) have been inundated with admissions, and the strain on resources is widely known. This is in part due to the sheer number of cases, but also due to the prolonged hospitalization period needed by critically unwell infected patients.

Wolverhampton, a populated city within the West Midlands region, received an early “peak” of cases [1], and a resultant early surge of admissions within the ICU. The finite capacity, limited resources, and anticipated prolonged respiratory wean has resulted in many different approaches in managing these patients within the ICU.

One approach to assist with the management of these patients was tracheostomy, with an aim to wean off sedation in order to facilitate eventual weaning from ventilation. Initial guidelines were limited as to an optimal time for this; a balance must be struck between early weaning and the risk of potential virus aerosolization and the subsequent risk of infection to the operator. We describe our early experiences with percutaneous tracheostomy in patients with COVID-19, highlighting safety precautions to prevent cross-infection to the operator.

## Technical report

During the period of March 2020 to April 2020, a total of 27 percutaneous tracheostomies were performed. All patients admitted to the ICU with COVID-19 were individually assessed for eligibility for tracheostomy. Criteria included: (1) COVID-19 confirmed on viral PCR nasal or throat swab; (2) intubated for at least seven days; (3) unsuccessful weaning while ventilated; and (4) coagulation status and platelet count within normal range. A formal referral was made to the on-call tracheostomy consultant for these patients, and a decision was made as a multi-disciplinary team.

A team brief was held as per the World Health Organization surgical safety guidelines [2]. The number of staff in the bed-space was limited to four where possible to reduce the exposure risk to the healthcare team. This included an airway handler (at the head of the patient), a tracheostomy operator (ENT surgeon or intensivist), and a senior assistant familiar with both the procedure and intensive care monitoring (including waveform capnography) with an ability to give intravenous medication if necessary. The final team member at the bed-space was responsible for operating the ventilator, including when on standby. A member of staff outside the bed-space was responsible for overseeing the procedure, providing help and situational awareness if needed.

All members of the team were equipped with powered air-purifying respiratory hoods (PAPR) and FFP3 masks, water-repellent suits, or surgical gowns and gloves in accordance with the Public Health England Guidelines [3]. The procedure was performed in a negative pressure single occupancy room where available, while following the UK National Tracheostomy Safety Project recommendations [4]. The required equipment was then set up and the relevant Intensive Care Society Checklist was then performed [5].

**Figure 1:**
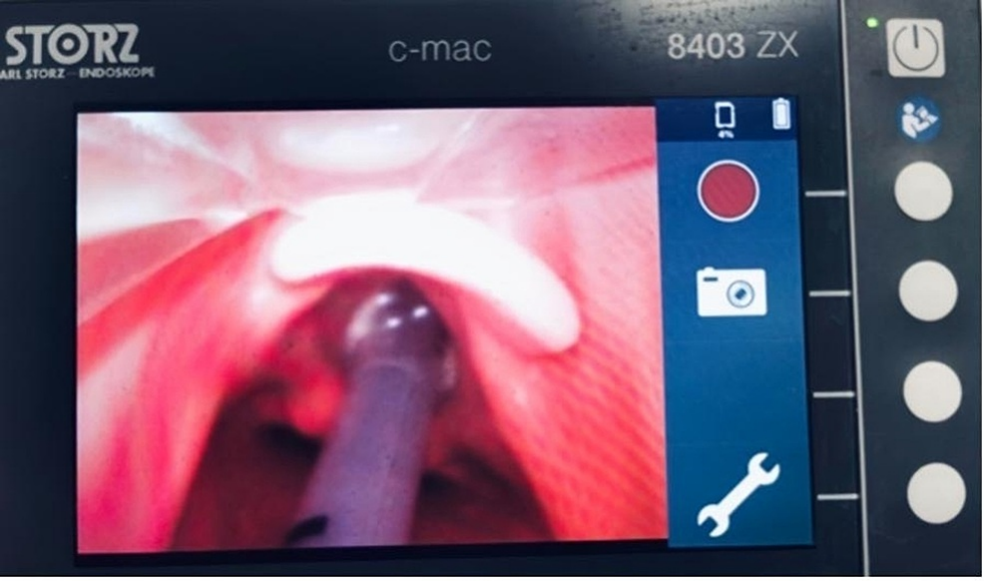
Endotracheal tube visualized by video laryngoscopy and withdrawn just distal to the vocal cords.

The patient is initially well sedated and a bolus of neuromuscular blockade is given prior to the procedure. A sterile transparent sheet is used to cover the patient’s face and upper chest during oropharyngeal suctioning. The patient is then pre-oxygenated with 100% oxygen for three to five minutes. The circuit used has a viral and bacterial heat and moisture exchange filter (HMEF) within the expiratory limb. Ventilation is then withheld by clamping the endotracheal tube (ETT) and putting the ventilator in standby mode. The ETT cuff is then deflated, and under videolaryngoscopy, it is withdrawn to be positioned distal to the vocal cords (Figure 1).

**Figure 2:**
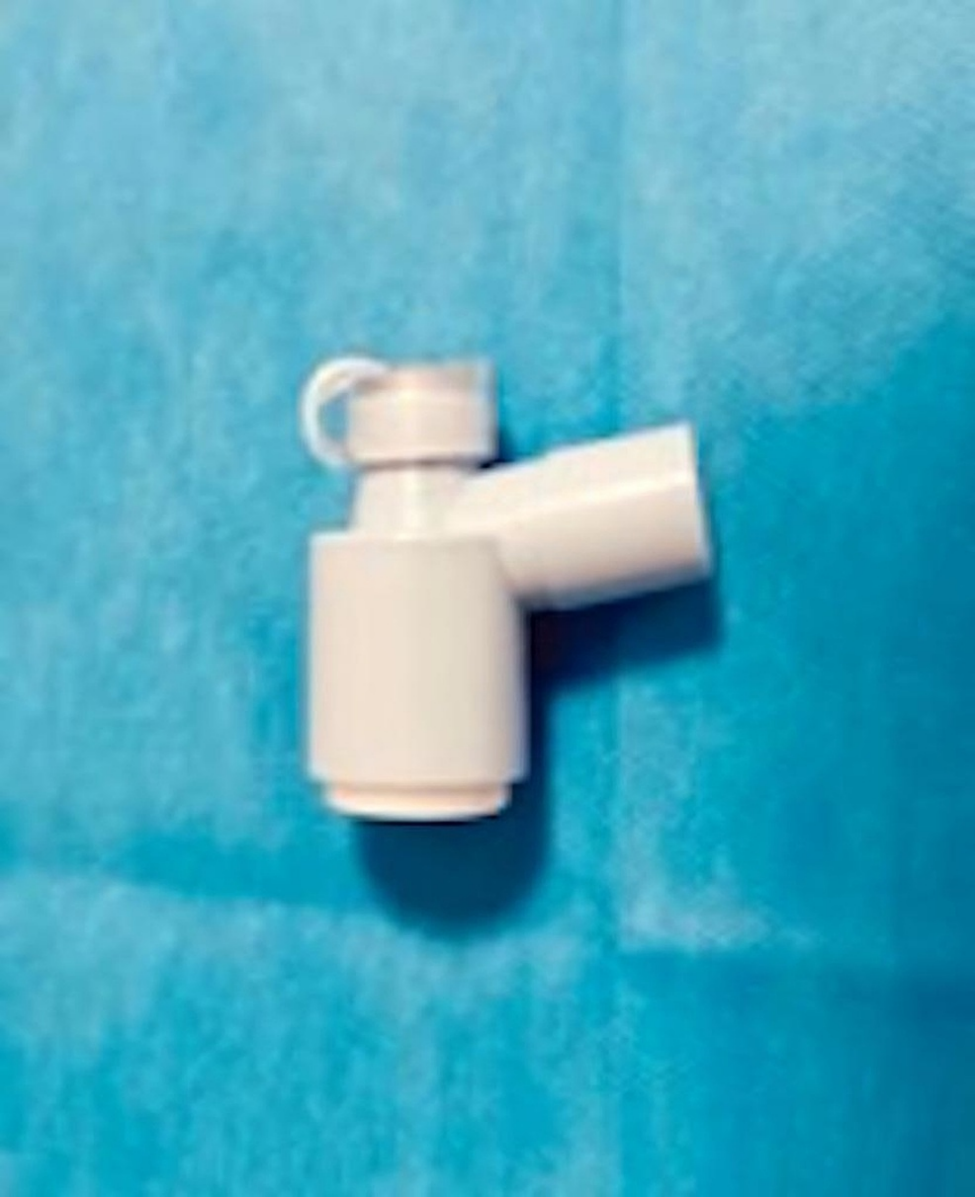
Swivel adaptor valve.

The cuff is then reinflated, the ETT is unclamped, and ventilation is allowed to resume. The patient is again pre-oxygenated. Then, with the ETT clamped and the ventilator placed in standby mode/turned off, a swivel adapter valve for a bronchoscope is attached (Figure 2).

The ETT is then unclamped and ventilation is recommenced in preparation to start the procedure. At this point, the skin of the anterior neck is prepared with 2% chlorhexidine and 70% alcohol solution and infiltrated with 4-5 mL of 1% lidocaine with 1:80,000 adrenaline.

**Figure 3:**
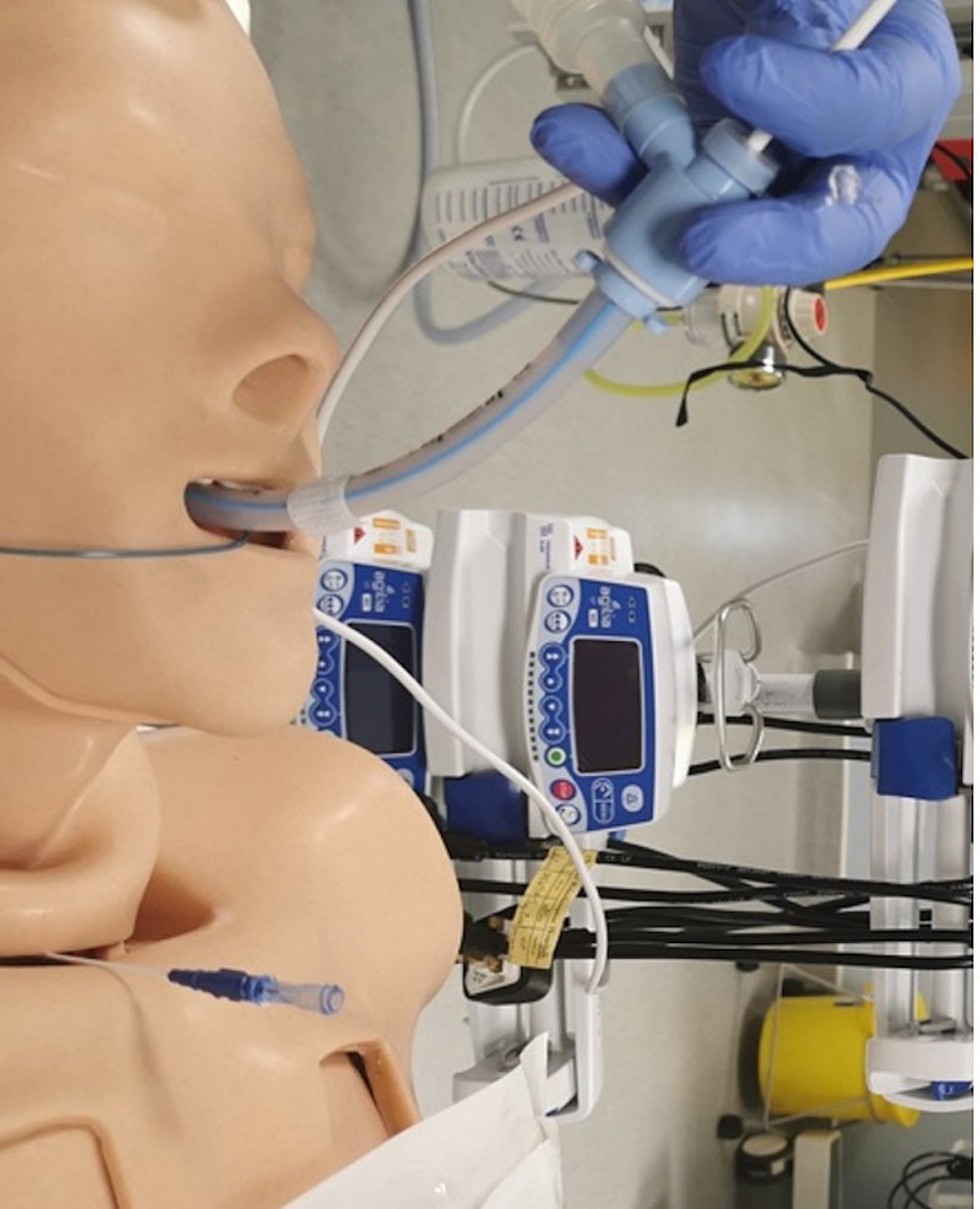
Tracheal puncture using cannulation needle. Note the bronchoscopy scope remains in situ, and the airway handler forms a tight seal around the swivel connector to minimize airway leakage.

**Figure 4:**
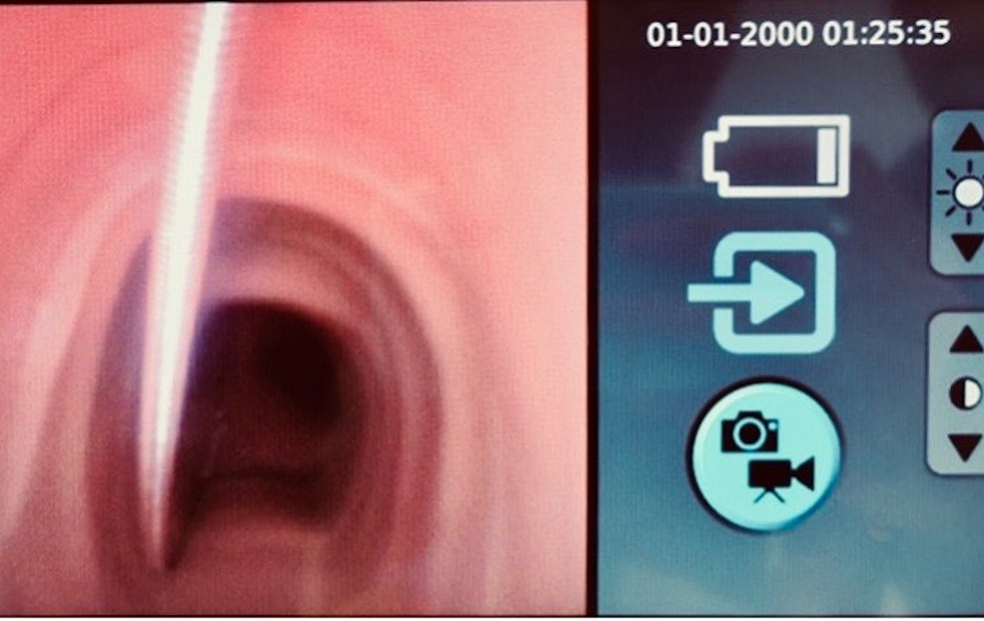
The guidewire should be clearly visualized with the bronchoscope.

The ventilator is put into standby mode and the procedure is commenced under direct bronchoscopy guidance while avoiding ventilation. This step should be performed as swiftly as is safely possible. Once the trachea is punctured and the guide-wire is inserted, the introducer sheath can be removed. This is demonstrated in Figures 3, 4.

**Figure 5:**
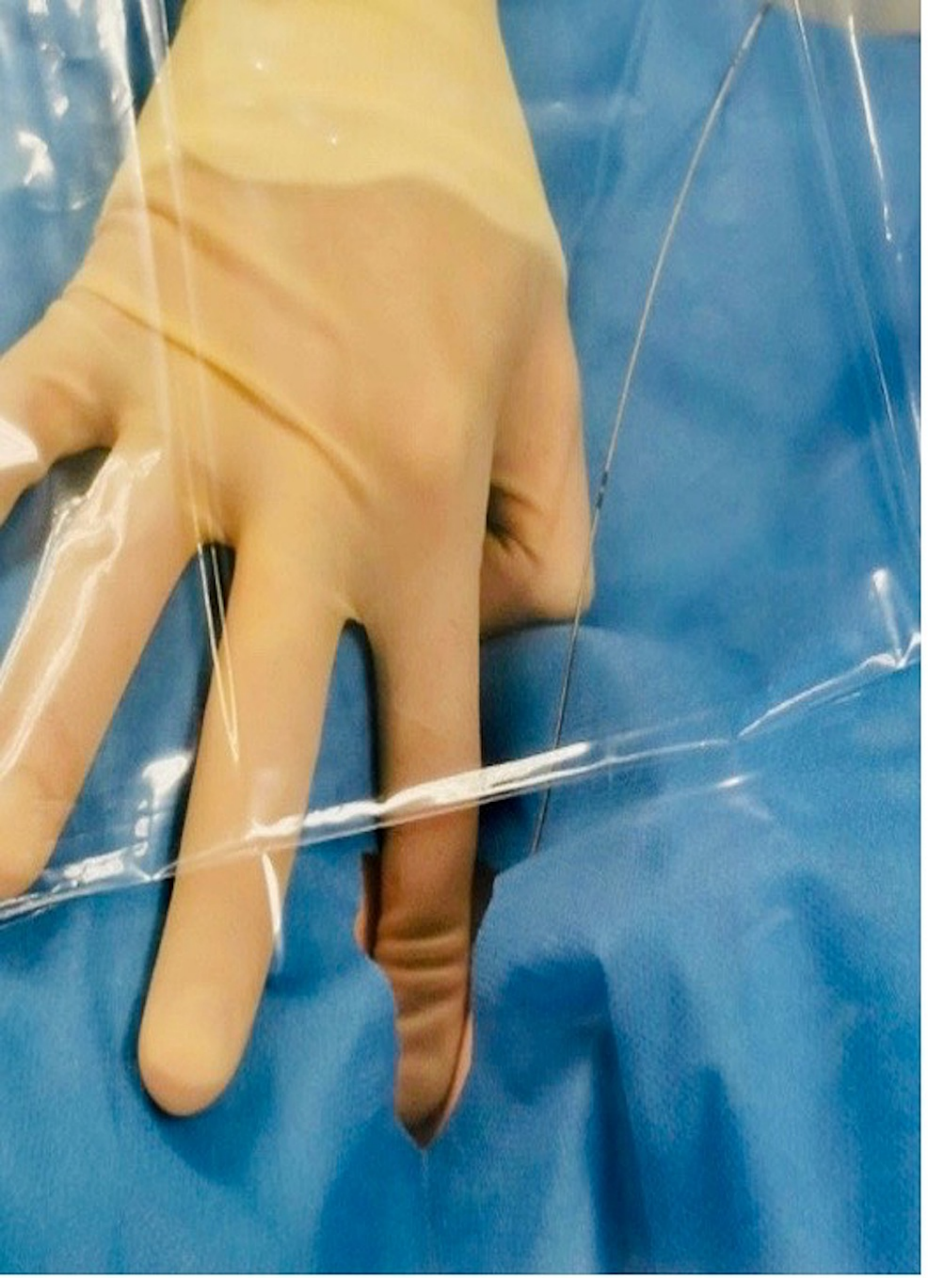
Manual covering of stoma site. If manual ventilation is required, the tracheal opening should be manually covered while maintaining sterility.

Should ventilation be required at this point due to low or dropping oxygen saturation, the operator should manually cover the tracheal opening with his/her finger around the guidewire with a sterile transparent sheet on top to minimize aerosol generation (Figure 5). Once safe to proceed, a small stab incision is made through the skin and the dilators swiftly inserted over the guidewire while ventilation is paused.

**Figure 6:**
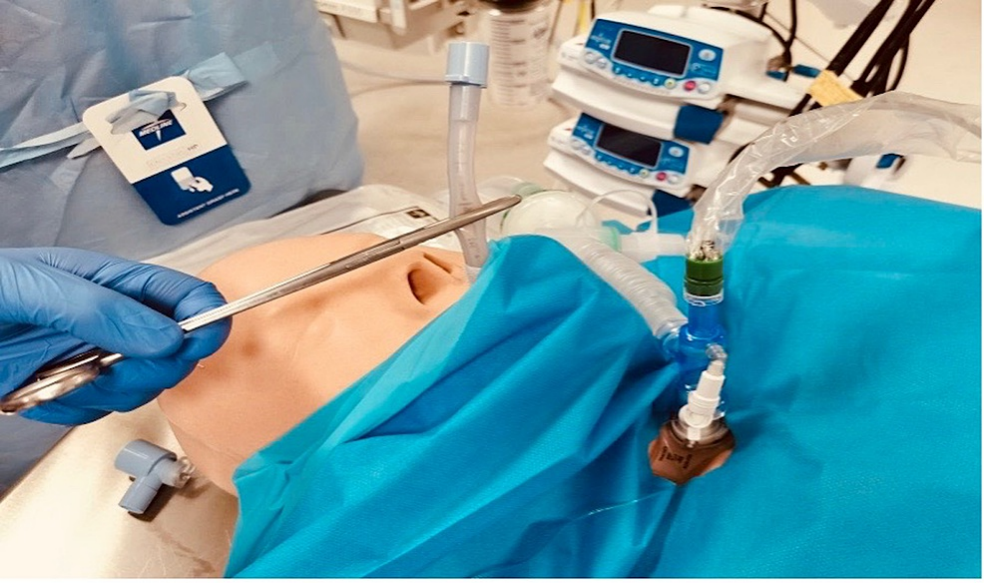
Connection to the ventilator circuit together with in-line suction.

Once the tracheostomy tube is inserted, the cuff is inflated, and a new circuit with an HMEF filter, in line suction, and nebulizer are attached to the tracheostomy (Figure 6). Ventilation is commenced via the tracheostomy only after the cuff is inflated, and confirmation is done with waveform capnography and visualization of chest rise, avoiding auscultation. The ETT can be removed and the tracheostomy tube secured appropriately. A post-procedural chest X-ray is then completed. All contaminated equipment is wrapped and immediately disposed into the appropriate clinical waste bin.

## Discussion

Early uncertainties regarding the management of COVID-19 patients in ICU have led to varied recommendations [4-6]. We describe our early experiences and adapted technique that benefits both the patient and optimizes operator safety.

Promotion of early wean with tracheostomy

It is widely known that prolonged periods of sedation is associated with increased mortality in the ICU setting. Other complications of prolonged periods of sedation may include hypotension, sarcopenia, gastrointestinal disturbances, and undernutrition. The wide range of complications is a large incentive to promote early wean from sedation and encourage weaning from mechanical ventilation, and this is largely facilitated by the insertion of a tracheostomy.

The prolonged need for ventilation in COVID-19 patients is likely due to the diffuse alveolar damage and interstitial inflammation [7], leading to extensive lung parenchyma damage and subsequent poor respiratory function. It therefore follows that these cohort of patients may suffer from the side effects of prolonged periods of sedation, and as such may have worse outcomes. A very early challenge during the COVID-19 pandemic was to balance the recognition of the need to wean sedation with the risk a tracheostomy may have to the operator.

In the 27 patients who underwent our technique, 17 were decannulated within 14 days, seven remained on pressure support ventilation, and three patients died due to causes unrelated to the early tracheostomy. This early success demonstrates favorable outcomes with tracheostomy, encouraging early building of respiratory muscle strength and promotion of early weaning from sedation.

Infectivity risk to the operator and team

Despite early successes, a balance must be struck between the promotion of early wean and risk of infection transmission to the operator during an aerosol generating procedure. Little was initially known about the infectivity and spread of severe acute respiratory syndrome coronavirus 2 (SARS-CoV-2). Early data suggest the “positive” cases may not necessarily correlate with “infectivity” [8]. This may be attributable to the decrease in viral load, with the peak (and subsequent infectivity) occurring approximately one to two days after symptom onset [9], and declining approximately eight days thereafter [10]. Our team performed percutaneous tracheostomies approximately seven days after ICU admission, or an estimated two weeks after symptom onset, at a theoretical point of a minimal viral load. To date, no operator or airway handler has been confirmed to have contracted SARS-CoV-2 from our procedures. This was recorded both in terms of recognized SARS-CoV-2 symptoms (fever, cough, shortness of breath, anosmia) or a positive nasopharyngeal swab on polymerase chain reaction.

To ensure minimal aerosolization and virus shedding to the team, we describe specific safety measures superadded to the ENTUK guidelines [4].

Minimal team members within the vicinity combined with effective pre-briefing avoid unnecessary exposure to non-essential staff. An “outside runner” facilitates only necessary equipment to be in the proximity of the procedure, mitigating the need for larger trolleys/trays of equipment within the area. We cover the patient at all times with a transparent sheet to reduce the risk of droplet spread.

We recommend pre-oxygenation prior to the airway handler withdrawing the ETT slightly for preparation of the tracheal window. Moreover, swivel connectors and disconnection of the circuit was only done after periods of pre-oxygenation to avoid patient desaturation. Minimal handling of the airway and multiple periods of oxygenation should minimize the time to desaturation and therefore avoid the need to hand ventilate the patient, potentially aerosolizing the virus. Adequate neuromuscular blockade was used both to facilitate the procedure and to minimize possible aerosolization or droplet spread, e.g., by inadvertent coughing. If ventilation is required, gentle, low-flow ventilation while occluding the stoma site was performed. All attempts must be continuously made to minimize virus aerosolization.

Recommendations of “enhanced PPE” [4] for both the operator and the airway handler have previously been made. We ensured this consisted of PAPR hoods together with an FFP3 mask underneath as contingency for hood failure during the procedure. The use of a sterile surgical gown over a fluid-repellent gown, and not in place of, ensures both sterility and safety of our operators.

## Conclusions

The early period of the SARS-CoV-2 pandemic was a time of great uncertainty in terms of operator safety and risk of infection. We describe a safe, consistent technique that has provided good outcomes both in terms of operator safety and patient outcomes. We emphasize clinician safety at all points during our technique, providing maximum protection at a time of significant risk.

We show that patients with SARS-CoV-2 should not be denied the benefits of an early tracheostomy as the safety risk to the tracheostomy team can be successfully mitigated by following precautions we advocate in this article.
